# Congruency of Information Rather Than Body Ownership Enhances Motor Performance in Highly Embodied Virtual Reality

**DOI:** 10.3389/fnins.2021.678909

**Published:** 2021-07-02

**Authors:** Ingrid A. Odermatt, Karin A. Buetler, Nicolas Wenk, Özhan Özen, Joaquin Penalver-Andres, Tobias Nef, Fred W. Mast, Laura Marchal-Crespo

**Affiliations:** ^1^Motor Learning and Neurorehabilitation Laboratory, ARTORG Center for Biomedical Engineering Research, University of Bern, Bern, Switzerland; ^2^Neural Control of Movement Lab, ETH Zurich, Zürich, Switzerland; ^3^Gerontechnology and Rehabilitation Group, ARTORG Center for Biomedical Engineering Research, University of Bern, Bern, Switzerland; ^4^Department of Neurology, University Neurorehabilitation, University Hospital Bern (Inselspital), University of Bern, Bern, Switzerland; ^5^Department of Psychology, University of Bern, Bern, Switzerland; ^6^Department of Cognitive Robotics, Delft University of Technology, Delft, Netherlands

**Keywords:** body ownership, embodiment, agency, incongruent information, motor performance, immersive virtual reality, multisensory information

## Abstract

In immersive virtual reality, the own body is often visually represented by an avatar. This may induce a feeling of body ownership over the virtual limbs. Importantly, body ownership and the motor system share neural correlates. Yet, evidence on the functionality of this neuroanatomical coupling is still inconclusive. Findings from previous studies may be confounded by the congruent vs. incongruent multisensory stimulation used to modulate body ownership. This study aimed to investigate the effect of body ownership and congruency of information on motor performance in immersive virtual reality. We aimed to modulate body ownership by providing congruent vs. incongruent visuo-tactile stimulation (i.e., participants felt a brush stroking their real fingers while seeing a virtual brush stroking the same vs. different virtual fingers). To control for congruency effects, unimodal stimulation conditions (i.e., only visual or tactile) with hypothesized low body ownership were included. Fifty healthy participants performed a decision-making (pressing a button as fast as possible) and a motor task (following a defined path). Body ownership was assessed subjectively with established questionnaires and objectively with galvanic skin response (GSR) when exposed to a virtual threat. Our results suggest that congruency of information may decrease reaction times and completion time of motor tasks in immersive virtual reality. Moreover, subjective body ownership is associated with faster reaction times, whereas its benefit on motor task performance needs further investigation. Therefore, it might be beneficial to provide congruent information in immersive virtual environments, especially during the training of motor tasks, e.g., in neurorehabilitation interventions.

## Introduction

In neurorehabilitation therapy, movements are often trained — e.g., with robotic support (Marchal-Crespo and Reinkensmeyer, 2009) — by visualizing the task in virtual reality (VR). VR has a great potential for neurorehabilitation, as it allows to mimic various activities of daily living in realistic or imaginary environments that can be adapted to the patients’ special needs, providing a motivating (Perez-Marcos et al., 2018), safe (Marchal-Crespo and Reinkensmeyer, 2008), and consistent environment (Rose et al., 2005). During conventional VR-based neurorehabilitation practice, the virtual environment (VE) is usually displayed on a computer screen. Within this two-dimensionally represented VE, patients interact via a symbolic virtual representation of their limbs (e.g., a cursor), drawing patients’ attention away from their real limbs (Wenk et al., 2019). Thus, the transfer of (re)trained skills into activities of daily living may be limited, because the movements and interactions trained in conventional VR-based neurorehabilitation interventions are far from those required in everyday life (de Mello Monteiro et al., 2014; Bezerra et al., 2018).

Within the last years, low-cost head-mounted displays (HMDs) have been made commercially available. HMDs allow a more realistic and immersive VR experience where the symbolic virtual representation of the users’ limbs may become a realistic self-representation (i.e., avatar) at a first-person perspective, promoting that the user “adopts” the virtual body and perceives it as becoming the own body. In VR, a sense of embodiment is experienced if the avatar’s body is — at least partly — processed like one’s own (artificial) body (Kilteni et al., 2012). Embodiment comprises three subcomponents: body ownership, agency, and self-location (Longo et al., 2008; Kilteni et al., 2012). Body ownership describes the feeling that the virtual body and/or its limbs are part of and belonging to oneself (Blanke, 2012) and results from the integration and interpretation of multimodal sensory information, e.g., visual and somatosensory signals (Botvinick and Cohen, 1998; Maravita et al., 2003; Ehrsson et al., 2004). Self-location describes the experienced location of the body in space (Blanke, 2012). Agency indicates the sense of initiating and being in control of one’s own actions (Braun et al., 2018).

Importantly, body ownership and the motor system share neural correlates, namely in frontal brain regions associated with motor control and movement planning (Wise, 1985; see also Tsakiris, 2010 for a review). For example, fMRI studies found that the bilateral activity in the ventral premotor cortex correlated with the strength of the feeling of ownership over a rubber hand (Ehrsson et al., 2004, 2005) and real own hand (Gentile et al., 2013). Additionally, support for a causal involvement of the premotor cortex in the feeling of body ownership was demonstrated. Dynamic causal modeling of EEG (Zeller et al., 2016) and fMRI data (Lee and Chae, 2016) showed that the connectivity between sensory (occipital and parietal) and premotor areas was influenced by the feeling of body ownership. Further, clinical literature implies that disorders in the feeling of body ownership are associated with deficits in frontal motor control. Stroke patients with lesions affecting connections to and from the ventral premotor cortex showed impaired ability to embody a rubber hand compared with healthy participants (Zeller et al., 2011). Altered processing in the premotor and somatosensory cortices was also associated with the feeling of disownership over own limbs in participants with body integrity identity disorder (van Dijk et al., 2013) and anosognosia, i.e., the denial of a diagnosed motor or sensory impairment of limbs in stroke patients (Berti et al., 2005). Taken together, a number of experimental studies in healthy and clinical population supports the notion that the feeling of body ownership relies on frontal brain structures known to be engaged in motor control and planning.

However, to date, less is known about the functional relevance of this neuroanatomical coupling between body ownership and the motor system. Previous studies suggest that body ownership may interact with motor performance (Grechuta et al., 2017, 2019; Shibuya et al., 2018a). In these studies, body ownership over an artificial/virtual hand is induced mostly through congruent visuo-tactile stimulation of the real and an artificial/virtual hand — i.e., rubber hand illusion (RHI; Botvinick and Cohen, 1998) or virtual hand illusion (VHI; Slater et al., 2008). Neuroimaging studies found enhanced activation in the motor system when participants observed spontaneous movements of an embodied virtual hand (Shibuya et al., 2018b). Moreover, this neural activation correlated with the reported feeling of body ownership, and participants tended to imitate the movements of the virtual hand, at least to initiate the movement. However, only a few behavioral studies have investigated if high vs. low levels of body ownership enhance motor performance. Grechuta et al. (2017) modulated participants’ body ownership over a virtual hand through congruent vs. incongruent visuo-tactile information — i.e., by a brush stroking the fingers — while participants performed a sensorimotor decision-making task. Whenever the brush stroked the index finger, participants had to press as fast as possible a button with the other (non-embodied) hand. Faster reaction times were associated with higher levels of body ownership. Further studies investigated the relationship between motor performance and body ownership in more complex motor tasks in VR. In Grechuta et al. (2019), authors measured performance in an air-hockey-like task in VR while either congruent or incongruent task-related auditive feedback was provided. Their results imply that incongruent auditive information led to worse motor performance and lower levels of body ownership. Shibuya et al. (2018a) altered the level of body ownership through visuo-motor feedback delays while participants draw a circle with a given speed. They observed that with an increasing visual delay, motor performance worsened, and body ownership decreased.

Together, the few studies investigating the potential benefits of embodiment on behavioral measures seem to support the notion of a functional link between body ownership and motor performance. However, none of the cited studies controlled for congruency of multisensory task-relevant information (Grechuta et al., 2017; Shibuya et al., 2018a). Previous studies modulated the level of body ownership by the congruency of multisensory stimulation; therefore, lower body ownership levels would be associated with the provision of incongruent information and higher levels of body ownership with congruent information — but not vice versa. This is an important limitation since exposition to incongruent, competing information may enhance task difficulty, for instance reflected in slower reaction times compared to congruent and unimodal information (e.g., MacLeod, 1991; Kerns et al., 2004). Hence, it remains unclear how congruency (or incongruency) effects confounded previous results on the influence of body ownership on motor performance. Furthermore, previous findings may also be limited because no motor tasks were employed (Shibuya et al., 2018b) or simple sensorimotor decision-making tasks were performed with the non-embodied hand (Grechuta et al., 2017). Finally, the assessment of body ownership varies across studies. Embodiment is usually assessed with questionnaires (e.g., Botvinick and Cohen, 1998; Grechuta et al., 2017, 2019; Shibuya et al., 2018a,b). However, standardized and validated scales barely exist. Longo et al. (2008) used a psychometric approach to identify how various items describing different subjective experiences related to embodiment are associated with its subcomponents. They revealed that an item commonly used to assess body ownership (Botvinick and Cohen, 1998; Lenggenhager et al., 2007; Petkova and Ehrsson, 2008; Slater et al., 2010; Grechuta et al., 2017) may rather be related to the location component. Further, objective metrics of embodiment were often not included in previous studies (e.g., Shibuya et al., 2018a,b). For example, galvanic skin response (GSR) has shown to be a valid method to measure the level of body ownership in an objective manner. GSR is a sensitive measure of implicit activation of the autonomous nervous system reflecting psychological arousal and has been shown to be modulated by the increased stress reaction when the embodied vs. non-embodied artificial or virtual limb is exposed to a threat (Armel and Ramachandran, 2003; Ferri et al., 2013; Grechuta et al., 2017, 2019).

In the present study, we investigated in 50 healthy participants the potential benefits of virtual body ownership on motor performance in a simple sensorimotor decision-making task with the non-embodied hand (Task 1) and a complex motor task performed with the virtual hand (Task 2). We aimed to modulate the level of body ownership without affecting agency or location. We provided congruent vs. incongruent visuo-tactile stimulation, i.e., participants felt a brush stroking their real fingers while seeing a virtual brush stroking the same vs. different virtual fingers. Importantly, to control for congruency effects, unimodal conditions were added to the experimental protocol. In these unimodal conditions with expected low body ownership, participants could either only see (but not feel) or only feel (but not see) a brush stroking their fingers. We hypothesize that participants in the congruent condition would experience a higher level of body ownership compared to the incongruent and unimodal conditions due to multisensory integration. We also expect a reinforced feeling of body ownership in the motor task compared with the decision-making task due to additional synchronous visuo-motor information. Moreover, we expect that the level of body ownership will be associated with faster reaction times (Task 1) and enhanced motor performance (i.e., higher accuracy and faster movements in Task 2) due to the neuroanatomical coupling of body ownership and the motor system. Finally, we hypothesize that participants in the congruent and unimodal conditions will be faster in the sensorimotor decision-making task and perform better in the motor task than in the incongruent condition, due to the absence of competing information.

## Materials and Methods

### Participants

A total of 56 healthy young participants were recruited from the campus of the University of Bern, Switzerland. Six participants had to be excluded due to data recording problems with the response box. The 50 participants (35 females, 15 males) included in the study were between 19 and 33 years old (*M* = 25.06, *SD* = 3.43). All participants were right-handed according to a short version of the Waterloo Handedness Questionnaire (Boucher et al., 1996), and reported normal or corrected-to-normal vision. Participants were naive to the hypotheses of the experiment and none reported to have participated in an RHI/VHI experiment before. The study was approved by the local ethics committee and all participants signed a written informed consent before starting the experiment. Participants were pseudo-randomly assigned to one of five experimental conditions, balanced for gender (7 females and 3 males per condition). Forty-four participants absolved the experiment in German, six in English. The used language was equally distributed across the conditions.

### Experimental Setup

#### Material

**FIGURE 1:**
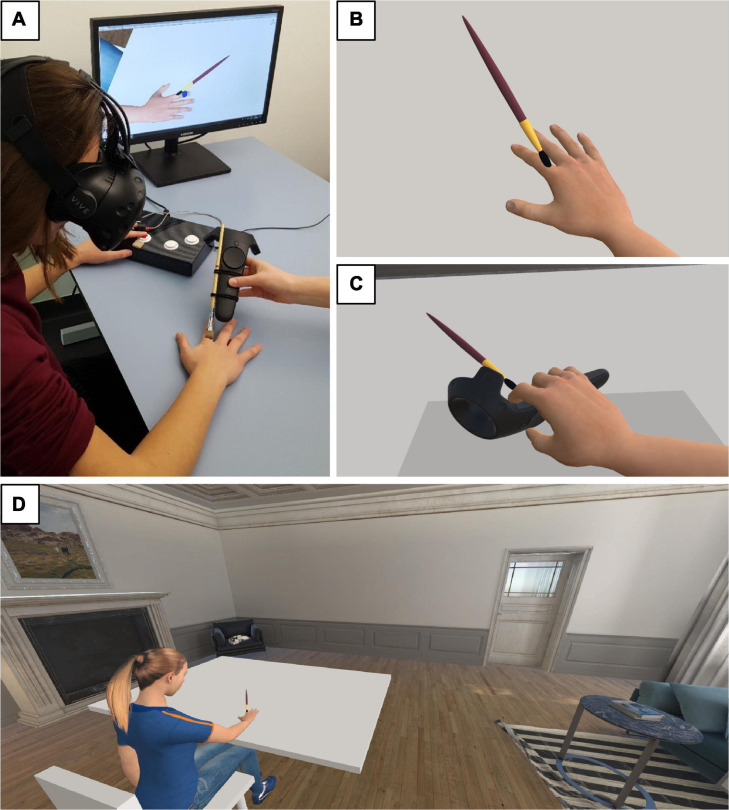
Experimental setup and VE. **(A)** A participant performing Task 1. Her index finger is stroked in a congruent mode and her left hand is on the response box. The blue dot seen on the virtual finger on the researcher’s screen indicates to the researcher which finger to stroke. By pressing the trigger button on the controller, the virtual brush appears. The virtual brush moves accordingly to the real brush attached to the controller and disappears as soon as the fingertip is reached. **(B)** Participant’s first-person perspective point of view in the VE during Task 1. The blue dot is not visible. **(C)** Participant’s first-person perspective point of view in the VE during Task 2. **(D)** Overview of the VE, including the female version of the avatar.

A head-mounted display (HTC Vive, HTC, Taiwan & Valve, United States) and two controllers (HTC Vive, Taiwan & Valve, United States) were employed in the VR setup. A 4-button response box (The Black Box ToolKit Ltd., United Kingdom) recorded the reaction times during the first experimental block and was used by the participants to answer the questionnaires in VR (Figure 1A). Galvanic skin response (GSR) was measured continuously during the whole experimental session with two electrodes attached to the ring and middle fingers of the left hand (g.Sensor, g.tec Medical Engineering GmbH, Austria).

#### Virtual Environment and Avatar

The VE was built in Unity game engine (version 2018.3.0f2; Unity Technologies, United States). A male and a female avatar were designed in MakeHuman, an open source software (version 1.1.1^1^). Participants saw the gender-matched avatar from a first-person perspective. During the first part of the experiment (Task 1, see section “Tasks and Experimental Procedure”), the avatar’s right arm was not animated (Figure 1B). In the second part (Task 2), participants received an HTC Vive controller to hold in their right hand (Figure 1C). The avatar’s right arm was animated by employing the position and orientation of the controller and using Unity’s Inverse Kinematics on the shoulder, elbow, and wrist. The avatar’s left arm was rendered in a way that the hand was located under the virtual table. Therefore, the avatar’s left arm was neither animated nor visible in the VE (Figure 1D). The VE consisted of a virtual living room (adapted from ArchVizPRO, Italy) but to minimize distractions, the participants were facing a virtual white wall during the tasks.

### Tasks and Experimental Procedure

#### Visuo-Tactile Information

Participants received visuo-tactile stimulation, i.e., they could feel and/or see a paintbrush stroking the fingers of the right virtual and/or real hand (Figure 1). The brush moved from the knuckle to the fingertip. To synchronize the stroking of the real and the virtual fingers, the real brush was attached to an HTC Vive controller. Pressing the trigger button of the controller led to the visual appearance of the virtual brush, which moved according to the real brush and disappeared as soon as the fingertip was reached. The virtual stroking lengths were scaled to the participant’s fingers lengths, which were measured before the experiment started. Every finger stroking trial had a duration of approximately 1.4 s, followed by a random time interval between 1 and 3 s before the next finger was stroked. The congruency and amount of sensory information (i.e., unimodal vs. multimodal) were modulated across five experimental conditions (see section “Experimental Conditions”).

#### Experimental Procedure

**FIGURE 2:**
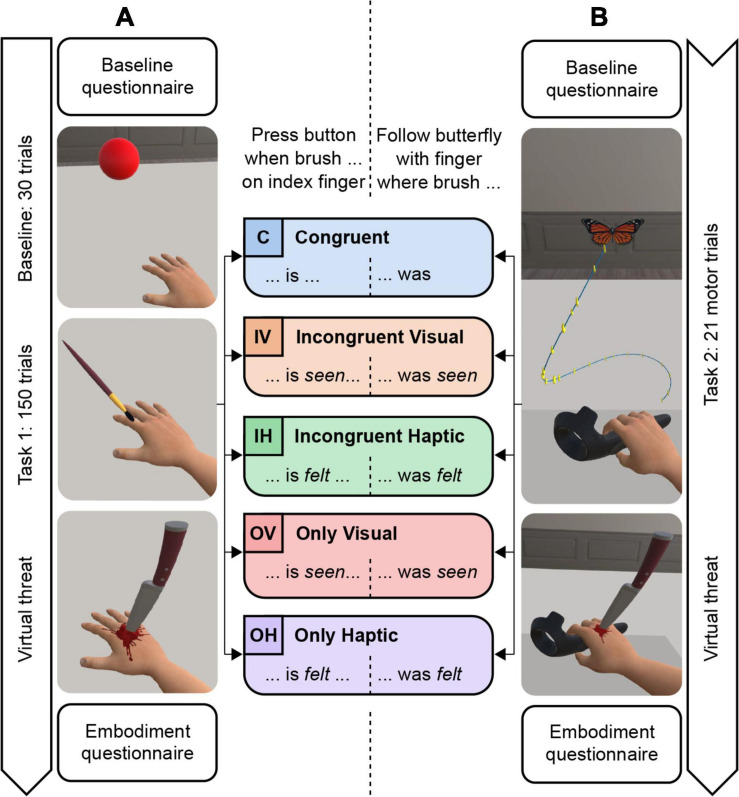
Experimental procedure and task instructions. **(A)** Block 1: Decision-making task. **(B)** Block 2: Motor task.

The whole experiment was completed in a single session of 55 min. The experiment consisted of two experimental blocks (Figure 2) during which participants performed two different tasks (Task 1: sensorimotor decision-making; Task 2: motor task, see below) while continuously being immersed in the VE with the HMD. Participants received visuo-tactile stimulation during Tasks 1 and 2, and additional visuo-motor synchronous feedback only during Task 2.

Before being immersed in the VE, participants were seated at a table with their right hand placed at a marked position in front of them to ensure a matching position between the real hand and the avatar’s hand. Participants were instructed not to move their right hand during the first block. Before starting the task, participants could visually explore the VE. After the first block, an HTC Vive controller was handed to the participants while the VE faded to black in order to not abruptly break the embodiment due to the change of the visual appearance of the avatar (see Figures 1B,C). Before receiving the controller, participants were presented with an image showing how to hold it to ensure a matching position between the real and the avatar’s hand (Supplementary Material 1). Participants were then instructed to move their right hand/arm with respect to the task instructions.

Both blocks started with a baseline questionnaire to assess the initial subjective level of body ownership (BA-Q; see Table 1). Directly after the tasks, a threat, i.e., a virtual knife, fell from above the vision field and stabbed the virtual hand. The knife and wound disappeared after 2.2 s. After a break of 60 s, participants answered the embodiment questionnaire (EM-Q; see Table 2). All participants performed the two blocks in the same order. We expected that the visuo-motor synchronous feedback in the motor task would additionally enforce the level of body ownership, and attempted to avoid confounds on the decision-making task. Task instructions (Supplementary Material 1) and questionnaires were presented in the VE to keep them standardized and to facilitate immersion. The items of the questionnaires appeared in a randomized order.

**TABLE 1:** Baseline Questionnaire (BA-Q).

**Body ownership**
Q1	It seems like the virtual hand is my hand.
Q2	It seems like the virtual hand is part of my body.
**Control items**
Q3	It seems like I have more than two hands.
Q4	It seems as if my real hand is becoming virtual.
**Distractors**
Q5	It seems as if the virtual living room is real.
Q6	It seems as if I am present in the virtual living room.
Q7	I like the virtual living room.
Q8	I feel comfortable in the virtual living room.

*Q1-4 (Longo et al., 2008); Q5-8 (self-generated).*

**TABLE 2:** Embodiment Questionnaire (EM-Q).

**Body ownership**
Q1	It seemed like the virtual hand was my hand.
Q2	It seemed like the virtual hand was part of my body.
Q3	It seemed like I was looking directly at my own hand.
Q4	It seemed like the virtual hand belonged to me.
**Location**
Q5	It seemed like my hand was in the location where the virtual hand was.
Q6	It seemed like the touch I felt was caused by the paintbrush touching the virtual hand.
**Agency**
Q7	It seemed like I was in control of the virtual hand.
Q8	It seemed like I was causing the movements I saw.
**Disownership/Loss of own hand**
Q9	It seemed like the experience on my real hand was less vivid than normal.
Q10	It seemed like I could not really tell where my real hand was.
**Control items**
Q11	It seemed like I had more than two hands.
Q12	It seemed as if my real hand was becoming virtual.
Q13	It seemed as if the virtual hand was drifting toward my real hand.
Q14	It seemed as if the virtual hand was controlling me.

*Q1-7, Q10-12 (Longo et al., 2008); Q8, Q13 (Kalckert and Ehrsson, 2012); Q9 (Bassolino et al., 2018); Q14 (Kalckert and Ehrsson, 2014).*

##### Block 1 (Task 1: sensorimotor decision-making task)

In Block 1, the participants first performed a Baseline task: pressing a button with their left (i.e., the non-embodied/non-visible) index finger as soon as a virtual red sphere appeared in front of them (30 trials). The purpose of this task was to account for interindividual differences in reaction times (Grechuta et al., 2017). Of note, no visuo-tactile stimulation was applied during the Baseline task.

In Task 1, participants were instructed to press a button with their left index finger as soon as their right (real or virtual) index finger was stroked (see section “Experimental Conditions,” Figure 2A, and Supplementary Material 1 for instructions). Task 1 consisted of 150 stroking trials of which the index finger was stroked in 30 trials. The visuo-tactile stimulation followed a pseudorandomized sequence which was computed individually for each participant and following the criteria (replicating Grechuta et al., 2017): (1) the sequence was structured in sets of five trials in which each finger was stroked once, (2) the same finger could not be stroked twice in a row, and (3) if two adjacent fingers were being stroked consecutively, the next finger could not be adjacent to either of them.

##### Block 2 (Task 2: motor task)

In Task 2, participants had to follow predefined paths visually presented in the three-dimensional space (Figure 2B). A motor trial consisted of following with the controller a path of stars that appeared as a butterfly flew away from the path start approximately 5 cm above the avatar’s hand. Seven different paths were presented three times each in a randomized order, resulting in a total of 21 motor trials.

Participants were instructed to follow the path with their right hand as accurately and fast as possible with the knuckle of the finger that was stroked when the butterfly appeared (see section “Experimental Conditions,” Figure 2B, and Supplementary Material 1 for instructions). The stars on the path disappeared when they were passed through with the correct finger. Additionally, participants were instructed to move continuously and only in a forward direction, even if they would miss stars. The speed of the flying butterfly was chosen so that it moved along each path in 1 s, which prevented participants to catch it before reaching the end of the path. The motor trial was completed as soon as the butterfly at the end of the path was touched with the correct finger and it disappeared together with the path. A green semitransparent sphere appeared at the beginning of Task 2 and after each path to indicate where the hand should be placed back on the table. The sphere disappeared as soon as the hand was in the correct position.

For the visuo-tactile stimulation, the number of fingers that were stroked between the motor trials was randomized between 4 and 14 fingers, with the only restriction that the same finger was never stroked twice in a row. As participants were holding the controller, the thumb was never stroked. Duration and interstimulus time interval of stroking trials were the same as in Task 1.

### Experimental Conditions

Participants stayed in the same experimental condition during both experimental blocks. Congruency and visuo-tactile information were modulated between five experimental conditions. In the congruent condition (C), the virtual brush stroked simultaneously the virtual equivalent of the finger that was stroked by the real brush and participants had to press the button as soon as their right index finger was stroked (Task 1) or follow the butterfly with the knuckle of the finger on which the brush was stroked (Task 2). In the congruent condition, the task instructions did not specify whether participants should react to the felt (real) or visually perceived (virtual) brush stroking (see Supplementary Material 1 for instructions). In the incongruent conditions, the virtual brush visually stroked another finger than the real brush, following the same pseudorandomized sequence as described above, and with the additional restriction that the virtual brush could not visually stroke the same finger as the real brush on the real hand. In the incongruent haptic condition (IH), the participants had to react to the haptic information applied by the real brush, i.e., press the button as soon as they felt that their right index finger was stroked/follow the butterfly with the knuckle of the finger on which they felt the brush. In the incongruent visual condition (IV), they had to react to the visual information provided by the virtual brush, i.e., press the button as soon as they saw that their virtual right index finger was stroked/follow the butterfly with the finger on which they saw the brush. In the only visual condition (OV), the participants could only see the virtual brush, but the real fingers of the participants were not stroked. Participants were instructed to press the button as soon as they saw that the virtual right index finger was stroked/follow the butterfly with the finger on which they saw the brush. In the only haptic condition (OH), participants could feel the real brush stroking their fingers, but no virtual brush was displayed. In this condition, participants had to press the button as soon as they felt that their right index finger was stroked/follow the butterfly with the knuckle of the finger on which they felt the brush.

### Measures

#### Body Ownership

##### Subjective body ownership

The subjective feeling of body ownership (BO) was measured at baseline at the beginning of both experimental blocks using two items (Q1 and Q2; BA-Q, Table 1) and after Task 1 and 2 using two additional items (Q1 – Q4; EM-Q, Table 2) from an established questionnaire (Longo et al., 2008). At baseline, only two BO items were included, to not bias participants toward body ownership. Additionally, the two items were masked by two control and four distractor items (BA-Q, Table 1). After Task 1 and 2, items assessing location, agency, and disownership, as well as control items were presented together with the body ownership items (EM-Q, Table 2). The total eight (BA-Q) and 14 (EM-Q) items, respectively, were rated on a 7-point Likert scale from −3 (strongly disagree) to 3 (strongly agree) with a “not answerable” option.

To quantify subjective body ownership at baselines and after Task 1 and 2, the mean of Q1 – Q2 and Q1 – Q4 were calculated, respectively (BO-Baseline; BO-Task). To assess how body ownership may change over time during the experiment, body ownership difference (BO-Diff) was defined as the mean of Q1 and Q2 at baseline subtracted from the mean of the same questions after Task 1 and 2.

Of note, item Q6 (“It seemed like the touch I felt was caused by the paintbrush touching the virtual hand.”), assigned to the subscale location according to Longo et al. (2008) was used to measure body ownership in the study conducted by Grechuta et al. (2017).

##### Objective body ownership

The objective level of body ownership was assessed by galvanic skin response (GSR) when the virtual limb was exposed to the virtual knife.

The continuous GSR data was cut into epochs with time windows from 10 s pre- to 10 s post-stimulus onset (knife appearance) for each participant and condition. The resulting time-series were interpolated to achieve a constant sampling rate of 20 Hz and filtered with a zero-lag fourth-order Butterworth low pass filter with a 5 Hz cut-off frequency. The 10 s before threat appearance were defined as baseline and the mean of the signal during this time window was subtracted at each time point from the data after stimulus onset.

Two different features were extracted from the GSR raw data: the GSR-peak, identified as the maximum value within 5 s post-stimulus; and the GSR-mean, defined as the average signal strength (i.e., integral of the curve) 5 s post-stimulus onset as GSR usually reach maximum values between 1 and 5 s after stimulus onset (Armel and Ramachandran, 2003; Ma and Hommel, 2013).

#### Task Performance

##### Reaction times (RTs; Task 1)

The RTs in Task 1 were computed as the time interval between the initialization of the stroking trial by the researcher and the participant’s button press on the response box, averaged for each participant and condition. To check for potential fatigue or training effects, mean RTs for Task 1 were binned and calculated for early, middle, and late trials (1/3 of the total amount of trials each).

To account for interindividual differences in reacting to stimuli, the baseline RTs were subtracted. Further, the first trial of the Baseline task and Task 1 were considered as familiarization trials and, therefore, disregarded from the analyses. Outlier trials (more than +/−2.5 SDs from participant’s RTs mean) were excluded.

##### Motor performance (Task 2)

Motor performance was quantified by calculating the accuracy and completion time in each motor trial in Task 2. The analyzed movements comprised the participant’s performed paths between the starting point of the defined path and the disappearance of the butterfly when the participant reached it at the end of the path. The overall accuracy was calculated as the mean absolute trajectory error between the participant’s movement trajectory and the defined path, and averaged over all trials per participant and condition — i.e., higher trajectory errors indicate lower accuracy. The completion time was calculated as the time between reaching the path starting point until the butterfly disappeared, and was averaged for each participant and condition. Since the paths had different lengths, the completion time was normalized with respect to the length of the corresponding path.

Sometimes participants did not catch the butterfly directly when reaching the end of the defined path, resulting in high trajectory errors (low accuracy) and a distorted SD of the corresponding participants. Therefore, trials were excluded if the trajectory error was higher than 1.5 times the interquartile range according to Tukey fences (Hoaglin, 2003). These identified outlier trials were disregarded from all analyses.

Further, to check for training effects or potential fatigue, mean accuracy and completion time were binned and calculated for early, middle, and late trials (1/3 of the total amount of trials each).

### Data Analysis

Statistical analyses were processed with Python 3.7.0 using the library SciPy 1.1.0 and additional packages from *R* 3.5.1. A significance threshold of α = 0.05 was chosen for all analyses. As all data fulfilled normality according to Kolmogorov-Smirnov’s test, and homoscedasticity according to Levene’s test, we used parametric tests. Where applicable, post-hoc pairwise *t*-tests were performed to compare levels of factors. If not otherwise stated, the Tukey method was used to correct for multiple comparisons.

#### Subjective Body Ownership and Agency

To analyze whether the reported mean levels of body ownership (BO-Baseline, BO-Task, BO-Diff), control items at baselines and after Task 1 and 2 (BA-Q and EM-Q), and agency (EM-Q after Task 2) differed across conditions, one-way ANOVAs with the between-subject factor Condition (Congruent (C), Incongruent Visual (IV), Incongruent Haptic (IH), Only Visual (OV), Only Haptic (OH) were computed.

#### Objective Body Ownership

Potential differences between conditions of the continuous GSR signal after the threat appearance were analyzed with 1-dimensional statistical parametric mapping (1D SPM; Pataky, 2012) with Python package spm1d 0.4. The GSR time-series data 10 s after the threat was considered as the dependent variable. SPM tests for differences across the time, correcting for multiple comparisons using Random Field Theory.

One participant whose GSR did not reach a threshold value of + or – 0.03 microSiemens within 5 s after threat in neither Block 1 nor Block 2 was defined as a non-responder and excluded from the GSR analyses of both blocks (Armel and Ramachandran, 2003). Additionally, participants with a GSR-mean 10 s post-threat window higher or lower than 2.5 SDs from all participants mean within the same condition were further excluded (e.g., Grechuta et al., 2017). This concerned one participant in Block 1.

#### Relationship Between Objective and Subjective BO

To study the relationship of subjective and objective body ownership, Pearson product-moment correlations between each participant’s questionnaire (BO-Task) and GSR measures (GSR-peak and GSR-mean) were computed.

#### Reaction Times (RTs) and Motor Performance

A one-way ANOVA with the factor Condition (C, IV, IH, OV, OH) was performed to investigate potential differences in RTs in the Baseline task across conditions. Two-way mixed ANOVAs with the between-subject factor Condition and the within-subject factor Trials (early, middle, late) were computed to analyze effects on performance measurements (RTs, accuracy, and completion time).

#### Relationship Between BO (and Agency) and Performance

To analyze how subjective body ownership (BO-Task and BO-Diff) was associated with overall RTs (Task 1) and overall motor performance measures (accuracy and completion time; Task 2), correlation analyses between each participant’s questionnaire and performance measure were computed using Pearson product-moment correlation coefficients.

Because of the strong correlation between BO-Task and agency across all conditions in the experimental Block 2 [*r*(48) = 0.45, *p* = 0.001], we additionally evaluated the correlation between the performance measures (accuracy and completion time) with the agency score.

Correlations were computed: (1) over all conditions, and (2) separately for incongruent conditions (IV, IH) and congruent/unimodal conditions (C, OV, OH).

#### Differences Across Experimental Blocks

Differences across the two experimental blocks for BO-Baseline and BO-Task were analyzed with a two-way mixed ANOVA with within-subject factor Block (Block 1, Block 2) and the between-subject factor Condition.

To analyze whether the overall RTs in Task 1 were associated with performance measures (accuracy and completion time) in Task 2, correlation analyses were computed using Pearson product-moment correlation coefficients.

## Results

Within this section, all statistical tests with *p* < 0.05 are reported. If there was a clear directed *a priori* hypothesis, additional one-sided significant statistical tests with *p* < 0.10 are reported. Non-significant statistical tests are listed in Supplementary Material 2.

### Block 1: Sensorimotor Decision-Making Task

#### Body Ownership

##### Subjective body ownership

**FIGURE 3:**
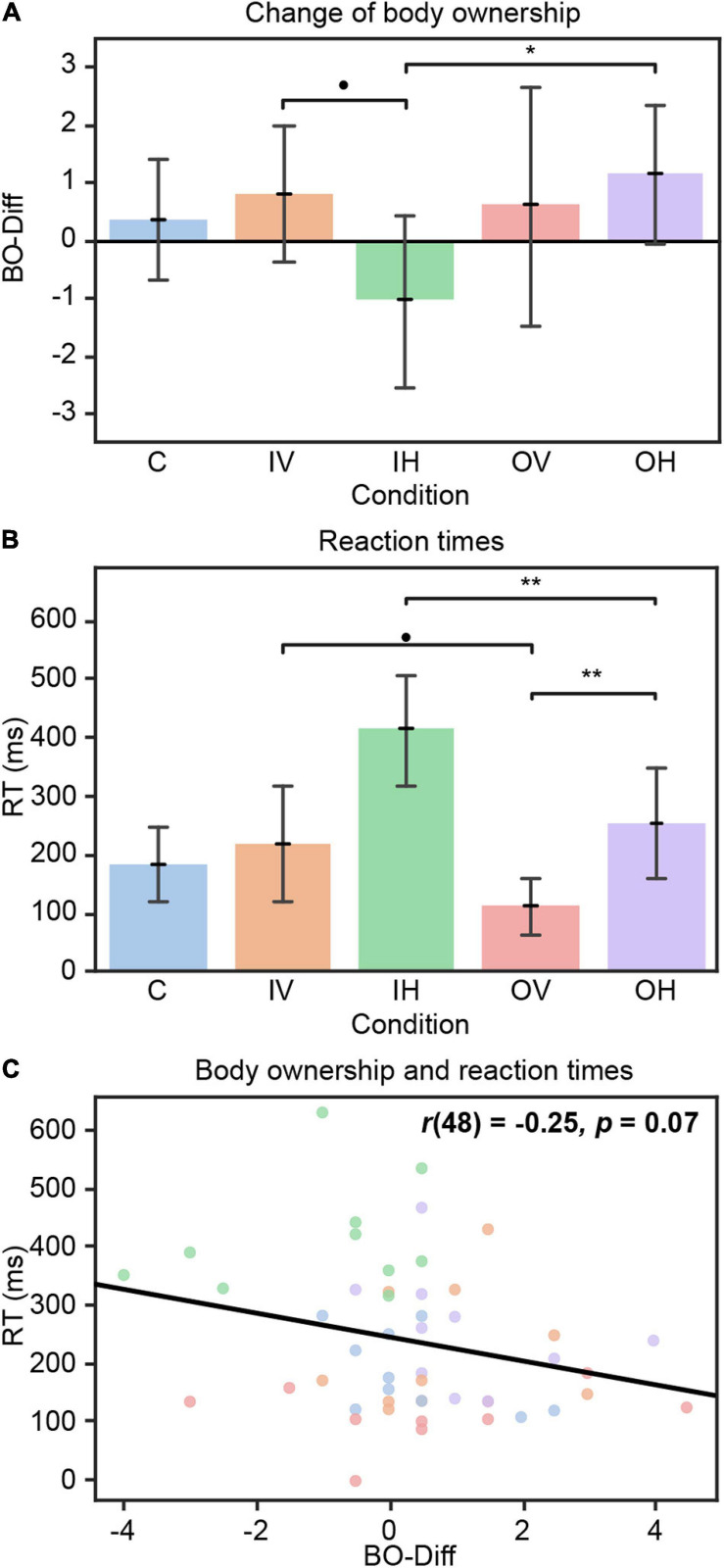
Results Block 1. **(A)** Change of body ownership from baseline to after Task 1 (BO-Diff) per condition. Positive values indicate an increase in subjective body ownership. **(B)** Mean reaction times over all trials of Task 1 per condition. The participant’s mean baseline value (from Baseline task) was subtracted from reaction times in Task 1. IH is significantly different from all other conditions. **(C)** Association of RTs during Task 1 and BO-Diff. C = congruent; IV = incongruent visual; IH = incongruent haptic; OV = only visual, OH = only haptic. Error bars represent standard deviation. • *p* < 0.10, *p < 0.05, **p < 0.01.

Regarding the reported body ownership (BO) values before the Baseline task (BO-Baseline), one-way ANOVA revealed a significant effect of Condition [*F*(4,45) = 2.59, *p* = 0.05], whereby pairwise comparisons of conditions did not differ significantly. No significant differences in body ownership after Task 1 (BO-Task) were found between conditions. In contrast, there was a significant main effect of Condition on the body ownership change from baseline to after Task 1 [BO-Diff; *F*(4,45) = 3.10, *p* = 0.02]. The level of BO in the OH condition increased more strongly than in the incongruent haptic (IH) condition [Figure 3A; IH – OH: *t*(45) = −3.24, *p* = 0.02].

There were no differences in the control items between conditions, neither at baseline nor after the tasks.

##### Objective body ownership

In the time window of 10 s after threat, differences of GSR between the conditions using SPM did not reach significance. Further, the objective (GSR-mean, GSR-peak) and subjective measures of BO (BO-Task) were not related.

#### Task Performance

No differences in reaction times between conditions were found in the Baseline task.

The two-way ANOVA revealed a main effect of Condition on the reaction times (RTs) during Task 1 [*F*(4,45) = 16.70, *p* < 0.001]. Participants in incongruent conditions showed slower RTs compared to the unimodal conditions of the corresponding modality [Figure 3B; IH – OH: *t*(45) = 4.13, *p* = 0.001; IV – OV: *t*(45) = 2.76, *p* = 0.06]. Further, conditions reacting on haptic stimulation were significantly slower than those responding on visual information [IH – IV: *t*(45) = 5.01, *p* < 0.001; OH – OV: *t*(45) = 3.65, *p* = 0.006]. Additionally, participants in the IH condition performed slower than those in C [C – IH: *t*(45) = −5.93, *p* < 0.001] and OV conditions [IH – OV: *t*(45) = 7.78, *p* < 0.001]. The lack of a main effect of Trial indicate that, across all conditions, participants did not improve or worsen from early to late trials. However, the significant Condition × Trials interaction effect [*F*(8,90) = 2.22, *p* = 0.03] revealed that participants receiving only haptic information provided slower RT over time [OH early – middle: *t*(90) = −2.44, *p* = 0.04, early – late: *t*(90) = −2.34, *p* = 0.05], while those receiving incongruent haptic information improved from early to middle trials [IH early – middle: *t*(90) = 2.66, *p* = 0.02].

#### Relationship Between Body Ownership and RTs

We found an association (one-sided significant) between RTs and the change of body ownership from baseline to after Task 1 (BO-Diff) [Figure 3C; *r*(48) = −0.25, *p* = 0.07]. This implies that an increase of self-reported body ownership from baseline to after the task goes in line with faster reaction times during Task 1. We did not find a correlation between overall RTs and BO-Task across all conditions.

### Block 2: Motor Task

The performance (accuracy and completion time) of one participant in the OH condition is higher than 2.5 SD from the condition’s mean. To avoid our results relying on this specific participant’s performance values, all analyses regarding performance measures in Task 2 were performed with and without this participant. If the significance of the results changed after the exclusion, both results are provided. Otherwise, only the results including all participants are reported.

#### Body Ownership

##### Subjective body ownership (and agency)

**FIGURE 4:**
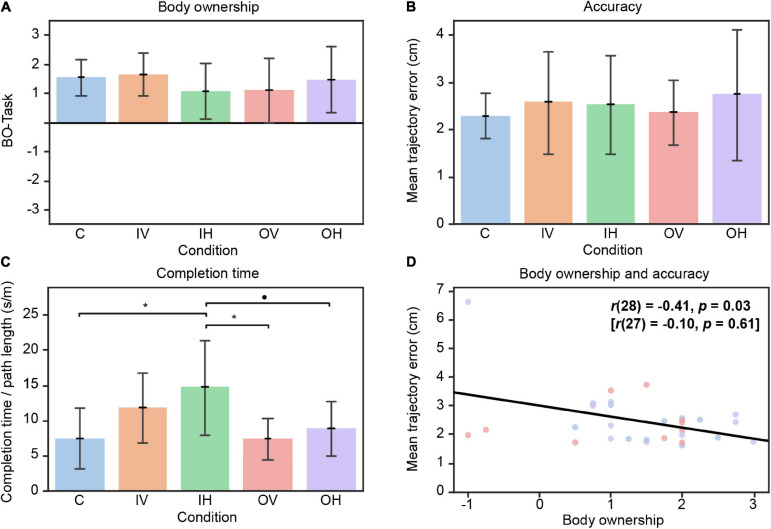
Results Block 2. **(A)** Means of the subscale body ownership (Q1–Q4) from embodiment questionnaire after Task 2 per condition. **(B)** Mean trajectory error over all trials of Task 2 per condition. Higher values indicate lower accuracy. **(C)** Mean completion time divided by the path length over all trials of Task 2 per condition. **(D)** Association of accuracy during Task 2 and BO-Task. Results in brackets represent data after removal of the outlier. C = congruent; IV = incongruent visual; IH = incongruent haptic; OV = only visual, OH = only haptic. Error bars represent standard deviation. • *p* < 0.10. *p < 0.05.

We did not find a main effect of Condition on BO-Baseline, BO-Task, and BO-Diff (Figure 4A). Additionally, participants reported statistically comparable levels of agency across all conditions.

There were no differences in control items at baseline (BA-Q). A main effect of Condition was found for the control items after task [EM-Q; *F*(4,45) = 2.66, *p* = 0.04]. However, post-hoc *t*-tests only revealed non-significant trends in differences between OV and IH - IV conditions.

##### Objective body ownership

No differences of GSR between the conditions were identified in the time window of 10 s after threat appearance. Further, BO-Task did not correlate with GSR-mean, nor with GSR-peak.

#### Motor Performance

For task accuracy, the two-way mixed ANOVA revealed a significant main effect of Trial [*F*(2,90) = 4.84, *p* = 0.01], but no main effect of Condition and no interaction effect. Post-hoc *t*-tests of the main effect showed that participants’ accuracy was significantly lower in the early trials compared to the late trials [Figure 4B; *t*(90) = 3.11, *p* = 0.007].

A main effect of Condition was found for task completion time [Figure 4C; *F*(4,45) = 4.08, *p* = 0.007]. In particular, participants in IH performed the task slower than participants in unimodal or congruent conditions [C – IH: *t*(45) = −3.28, *p* = 0.02; IH – OH: *t*(45) = 2.64, *p* = 0.08; IH – OV: *t*(45) = 3.27, *p* = 0.02]. No main effect of Trial and no interaction effect between Trial and Condition were found in task completion time.

Finally, the two measurements of motor performance (accuracy and completion time) were not related across all participants.

#### Relationship Between Body Ownership and Agency With Motor Performance

In conditions perceiving congruent or unimodal information (C, OH, OV), BO-Task (but not BO-Diff) was correlated with accuracy [Figure 4D; *r*(28) = −0.41, *p* = 0.03]. The higher the self-reported level of body ownership after the motor task was, the higher the accuracy. Moreover, there were trends toward a correlation between agency and accuracy [*r*(28) = −0.36, *p* = 0.05] and between agency and completion time [*r*(28) = −0.36, *p* = 0.05] in congruent and unimodal conditions.

However, after removing the potential outlier in the OH condition, the correlations lost significance [BO-Task – accuracy: *r*(27) = −0.10, *p* = 0.62; agency – accuracy: *r*(27) = −0.00, *p* = 0.99] except for a trend for agency being associated with task completion time [*r*(27) = −0.32, *p* = 0.09].

When taking into account all conditions and only incongruent conditions (IV, IH), neither body ownership (BO-Task/BO-Diff) nor agency were linked with the measurements of motor performance.

### Analyses Across Blocks

#### Body Ownership

For body ownership during baseline (BO-Baseline), the two-way mixed ANOVA revealed a significant main effect of Block [*F*(1,45) = 14.47, *p* < 0.001], but no main effect of Condition and only a trend in the interaction effect between Block and Condition. Post-hoc comparison revealed a significantly higher subjective body ownership level at baseline of Task 2 compared to baseline of Task 1 [*t*(45) = −3.80, *p* < 0.001].

For body ownership after tasks (BO-Task), analyses revealed a main effect of Block [*F*(1,45) = 12.27, *p* = 0.001], but no main effect of Condition and interaction effect. Post-hoc comparison revealed that body ownership after the motor task (Task 2) was significantly higher than after the sensorimotor decision-making task (Task 1) across all conditions [*t*(45) = −3.5, *p* = 0.001].

#### Task Performance

Participants providing faster reaction times (RTs) in Task 1 also showed faster completion times in the motor trials of Task 2 [*r*(48) = 0.44, *p* = 0.002]. In contrast, RTs in Task 1 were not associated with accuracy in Task 2.

## Discussion

The present study aimed at investigating if an increase in body ownership over an avatar in an immersive virtual reality training is associated with improved motor task performance. Further, we aimed at identifying potential confounds introduced by modulating embodiment using multisensory stimulation — namely, congruency effects — on motor performance. Fifty healthy young participants experienced the virtual hand illusion (VHI) while performing a sensorimotor decision-making and a motor task in immersive virtual reality. Both tasks were embedded in visuo-tactile stimulation modulating the congruency and amount of sensory information.

### Congruency of Information Enforces Reaction and Completion Times

To disentangle potential confounds between body ownership induction and congruency of information on motor performance measures, we controlled for congruency effects by adding unimodal conditions with hypothesized low body ownership. We expected that participants in the congruent and unimodal conditions would be faster in the sensorimotor decision-making task and perform faster and more accurately in the motor task compared to participants in the incongruent conditions, due to the absence of competing information.

In line with our hypothesis, we found congruency effects on reaction times and motor performance measures. In the decision-making task (Task 1), reaction times in unimodal conditions (only visual and only haptic) were faster than in incongruent conditions of the corresponding sensory modality, especially in the haptic modality. Similarly, incongruency of visuo-tactile information was associated with slower performance in the motor task (Task 2): participants in the incongruent haptic condition needed more time to complete the task than participants in the unimodal and congruent conditions. Our results speak for a general improvement of motor performance due to congruency effects, as the enhanced speed did not come at the cost of decreased accuracy: participants in unimodal/congruent and incongruent conditions all increased the accuracy during the motor task (i.e., no interaction effects between condition and trials were found). Reaction and completion times may more closely reflect mental processing speed than task accuracy — a variable previously shown to be highly sensitive for congruency effects (e.g., Jensen and Rohwer, 1966; Naber et al., 2016).

Further, modality effects (i.e., visual vs. haptic conditions) may have also affected the reaction times and motor performance measures across conditions. Participants in incongruent and unimodal conditions reacting to visual information were faster than participants in the corresponding conditions responding to haptic information. This is in line with research on the visual dominance effect, demonstrating that visual stimuli are prioritized over and faster processed than haptic and auditory stimuli during bimodal presentation in healthy adults (e.g., Hecht and Reiner, 2009). Body ownership over an artificial/virtual limb may promote visual dominance to resolve multisensory conflicts. In line with this notion, inducing body ownership illusion has been associated with an increased weighting of visual information and attenuation of tactile information (Zeller et al., 2016; Isayama et al., 2019). This could explain why in our sample with generally high body ownership levels, participants in the incongruent visual condition were not slower than participants receiving congruent or only visual information, as opposed to the results of Grechuta et al. (2017). Potential incongruency effects may have been minimized due to the enhanced attention toward the visual feedback. However, it is important to note that we did not control for the timing between the visual appearance of the brush and the felt brush stroking. It relied on the precision of the researcher to press a button exactly when touching the finger to initiate the visual finger stroking. Therefore, different timing across modalities could confound the results.

Together, we partially confirm our hypothesis on an improved performance due to congruency effects. We found that participants in the unimodal conditions are faster in the sensorimotor decision-making task and participants in the congruent/unimodal conditions perform better in the motor task than in the incongruent condition due to the absence of competing information. Yet, and contrary to our expectations, no congruency effects were found for task accuracy. It is possible that participants performed rather quickly at the expense of accuracy. Thus, in our immersive virtual reality training, it is likely that congruency of information is the modulatory variable accounting for differences in reaction times and performance measures shown without different levels of body ownership across conditions. Our results highlight the possibility that previous studies reporting a link between body ownership and behavioral measures may be confounded by congruency effects that were induced by the body ownership illusion.

### Body Ownership and Agency Are Associated With Better Motor Task Performance in Congruent and Unimodal Conditions

We hypothesized that, due to the neuroanatomical coupling between body ownership and the motor system, an increase in body ownership may be used as a tool to boost motor performance. Therefore, we performed correlation analyses to study the relationship between the reaction times and motor performance measures with reported body ownership across conditions.

For the sensorimotor decision-making task (Block 1), we found an association between the change of body ownership after the task with the reaction times. Thus, we replicate previous findings (Grechuta et al., 2017), but differently to previous studies, we show that an increase in body ownership during the task rather than the level of body ownership after the task may facilitate sensorimotor decision-making. For the motor task with the embodied virtual hand (Block 2), body ownership and motor performance were correlated (*p* = 0.03), suggesting increased task accuracy with higher levels of body ownership if participants received unimodal or congruent information. However, after the removal of one outlier participant, this correlation did not remain.

Facilitated reaction times with increased body ownership have previously been discussed to support the theory on a functional coupling between motor brain areas and brain areas involved in embodiment. According to this theory, body ownership is not considered to be an exclusively perceptual and/or subjective multimodal state but tightly coupled to systems involved in decision-making and motor control (Grechuta et al., 2017). Grechuta et al. (2017, 2019) propose that body ownership is a by-product of consistent motor predictions and feedback, underlining a bidirectional effect of body ownership and performance. The higher the experienced level of body ownership is, the better participants may plan and perform their movements. In turn, the more accurately participants perform the task, the higher may be the experienced level of body ownership.

Further, we found significant correlations between agency and completion time and task accuracy, i.e., a higher level of reported agency was associated with faster movements and higher accuracy for congruent and unimodal conditions. The experienced agency over a movement may also depend on motor predictions and feedback. By comparing the predicted sensory outcomes of a motor command with the actual feedback, a match leads to the feeling of being the actor of the performance and, therefore, agency (Braun et al., 2018). This additionally supports a bidirectional influence of motor performance and embodiment.

However, it needs to be noted that our results on the motor task (Block 2) are mainly explained by the data of one participant who reported low levels of body ownership and agency and presented lower tasks skills, i.e., more inaccurate and slower task performance than the rest of (more-skilled) participants. Body ownership and agency were not associated with task performance in these relatively high skilled participants. One explanation could be that the influence of embodiment on motor performance may critically depend on the participant’s skill level. Further, ceiling effects in the performance may have canceled out small differences across body ownership in the highly skilled group.

Further, another factor to consider are technical limitations of HMDs. HMDs present a display lag, i.e., there is a difference in the time between the participant’s head movement and the generated change in the visual scene rendered on the HMD. These display lags — noticeable by the user even at short durations (< 20 ms) — were found to be associated with reported severity of cybersickness (e.g., Feng et al., 2019; Palmisano et al., 2020). Cybersickness refers to a constellation of adverse effects often experienced during VR exposure, such as oculomotor discomfort, disorientation or nausea (e.g., Palmisano et al., 2017). Further, the decreased compatibility between visual and non-visual sensory information (e.g., proprioceptive) induced by display lags was also found to be associated with reduced experiences of presence (e.g., Weech et al., 2019; Kim et al., 2020). In VR literature, the sense of presence is most commonly defined as the subjective experience of “being there” in the virtual environment, as opposed to physically being situated in the “real world” (e.g., Witmer and Singer, 1998; Skarbez et al., 2017). Therefore, display lags may have raised discomfort in our participants and reduced their sense of presence in the VE, limiting potential beneficial effects of embodiment on task performance. Display lags may have especially impacted the performance in the motor task, as in this task, head movements were more likely carried out in order to follow the paths than in the decision-making task, where participants had to monitor the finger stroking. Future studies aiming at quantifying display lags may help to disentangle such confounds, for example, the difference in the participant’s virtual and physical head orientation (DVP) has recently been suggested as a reliable measure to predict perceptual and psychophysiological consequences of multisensory conflicts in VR (Kim et al., 2020; Palmisano et al., 2020).

Interestingly, incongruency of information seems to prevent an association of body ownership and agency with motor task performance. While a high level of embodiment may be beneficial for motor performance in congruent stimulation due to an enforced matching between perceived (virtual) and executed movements, high levels of embodiment could have a detrimental effect when the received stimulation is incongruent; the higher the experienced level of embodiment over the virtual hand, the stronger may be the mismatch between the perceived (virtual) and executed movements, hampering performance. This interpretation is in line with previous studies showing that motor performance was more prone to interference effects (induced by incongruence between the seen and the performed movement) when the seen virtual hand was perceived as part of one’s own body (Burin et al., 2019).

Together, our results suggest that enforcing body ownership along with agency might be beneficial for motor task performance if the received information is congruent. However, further investigations are needed, especially with less-skilled participants or challenging motor tasks.

### First-Person Perspective Immersive Virtual Reality Results in a High Level of Body Ownership

We found high subjective levels of body ownership across all conditions, i.e., independent of the visuo-tactile congruency of information. Against our hypothesis, the subjective rating of body ownership — assessed by means of an established body ownership subscale (Longo et al., 2008) — after the tasks did not differ between conditions. It is possible that ceiling effects may have diminished potential differences in body ownership across conditions since our experimental setup enforced body ownership by fulfilling several criteria known to top-down increase body ownership (Tsakiris, 2010): (i) first-person perspective virtual reality (Slater et al., 2010; Petkova et al., 2011), (ii) realistic appearance (Maselli and Slater, 2013) and posture of the virtual limb (Tsakiris and Haggard, 2005; Pritchard et al., 2016), and (iii) virtual limb connected to the body (Perez-Marcos et al., 2012). Thus, our findings are in line with previous notions stating that visuo-tactile stimulation might not be necessary to induce and/or enhance body ownership in first-person perspective immersive virtual reality (Maselli and Slater, 2013; Kokkinara and Slater, 2014; Rubo and Gamer, 2019). Nevertheless, haptic stimulation may have other favorable effects for motor learning and neurorehabilitation — e.g., for proprioception training (Cuppone et al., 2016), to enhance somatosensory information during neurorehabilitation (Gassert and Dietz, 2018; Özen et al., 2021), or to promote more variable tasks during training (Basalp et al., 2019).

Further, and in line with our hypothesis, subjective body ownership levels were higher after the motor task (Block 2) than the decision-making task (Block 1). It is likely that visuo-motor synchronies additionally enforced body ownership independently of the conditions (Sanchez-Vives et al., 2010), notably already at baseline. When entering the second experimental block, participants tested the movement of their virtual arm before motor task performance, resulting in significantly higher subjective body ownership levels at baseline of Block 2 than Block 1. Alternatively, carry-over effects from Block 1 are also possible as participants stayed immersed in the VE during the whole experiment.

Finally, the assessment of body ownership may critically depend on the measures used. When comparing questionnaire item Q6 “It seemed like the touch I felt was caused by the paintbrush touching the virtual hand” — previously used to assess body ownership by the group of Grechuta et al. (2017) – we found significantly higher values in the congruent versus incongruent conditions [C – IH: *t*(27) = 3.39, *p* = 0.006, C – IV: *t*(27) = 2.67, *p* = 0.03], replicating previous findings (Grechuta et al., 2017). However, this item is thought to reflect the “location” dimensionality of embodiment rather than “body ownership” (Longo et al., 2008). Further, this item may confound congruency effects of visuo-tactile stimulation with the feeling of body ownership.

In addition to the questionnaires, we implemented GSR as an objective measure of body ownership. In contrast to previous studies, we did not find associations with questionnaire-based values (Armel and Ramachandran, 2003; Ferri et al., 2013; Grechuta et al., 2017). This is in line with the finding that experienced differences in body ownership may not be captured by the GSR when the virtual limb is exposed to a threat, as this may trigger a direct affective response that is independent of body ownership, and instead, neutral impacts should be preferred (Ma and Hommel, 2013).

Together, our results suggest high top-down induction of body ownership over avatars in first-person perspective immersive VR that is additionally enforced by visuo-motor synchronies of the task. Further, our results imply that questionnaires assessing body ownership should be selected carefully. Previous findings on the influence of body ownership on motor performance may have been confounded by the questionnaires used and congruency effects across conditions.

### Study Limitations

The findings of our study must be interpreted within the frame of several limitations. First, the brush stroking of the fingers was manually initiated and performed by the researcher. Even though the same (trained) researcher performed the stroking — and visual feedback was provided on a computer screen to indicate the correct timing — we did not control for the exact brush stroke onset and stroking duration across conditions. Second, we did not assess a baseline of motor performance in the motor task, and therefore, some participants might have been more skilled than others, regardless of being pseudo-randomly assigned to the five conditions.

## Conclusion

The goal of this study was to understand and disentangle how body ownership and congruency of multisensory information interact with motor performance in virtual reality. Our results suggest that VR-based motor tasks providing congruent (multi)sensory feedback and enforcing body ownership and agency via visuo-motor synchronies may best support motor training. The use of first-person perspective immersive VR may simplify the implementation of efficient training environments in (robotic) neurorehabilitation, as they strongly enforce virtual embodiment independently of congruency of visuo-tactile information.

## Data Availability Statement

The dataset presented in this study can be found online in the following repository: doi: 10.5281/zenodo.4877633.

## Ethics Statement

The studies involving human participants were reviewed and approved by the Cantonal Ethics Committee Bern. The patients/participants provided their written informed consent to participate in this study.

## Author Contributions

IO, KB, and LM-C designed the study and wrote the manuscript. IO, NW, ÖÖ, and JP-A set up the experiment. NW programmed the motor task and contributed to the control of the avatar. IO tested all subjects and analyzed the data. NW and JP-A contributed to the analysis of kinematic data. ÖÖ contributed to the analysis of physiological data. All authors edited and revised the manuscript and approved the submitted version.

## Conflict of Interest

The authors declare that the research was conducted in the absence of any commercial or financial relationships that could be construed as a potential conflict of interest.

## Footnotes

1 http://www.makehumancommunity.org

## References

[B1] Armel K. C. Ramachandran V. S. (2003). Projecting sensations to external objects: evidence from skin conductance response. *Proc. Biol. Sci.* 270 1499–1506. 10.1098/rspb.2003.2364 12965016 PMC1691405

[B2] Basalp E. Marchal-Crespo L. Rauter G. Riener R. Wolf P. (2019). Rowing simulator modulates water density to foster motor learning. *Front. Robot. AI* 6:74. 10.3389/frobt.2019.00074 33501089 PMC7806073

[B3] Bassolino M. Franza M. Bello Ruiz J. Pinardi M. Schmidlin T. Stephan A. (2018). Non-invasive brain stimulation of motor cortex induces embodiment when integrated with virtual reality feedback. *Eur. J. Neurosci.* 47 790–799. 10.1111/ejn.13871 29460981 PMC5900900

[B4] Berti A. Bottini G. Gandola M. Pia L. Smania N. Stracciari A. (2005). Shared cortical anatomy for motor awareness and motor control. *Science* 309 488–491. 10.1126/science.1110625 16020740

[B5] Bezerra ÍM. P. Crocetta T. B. Massetti T. da Silva T. D. Guarnieri R. de Meira C. M. (2018). Functional performance comparison between real and virtual tasks in older adults. *Medicine (Baltimore)* 97:e9612. 10.1097/MD.0000000000009612 29369177 PMC5794361

[B6] Blanke O. (2012). Multisensory brain mechanisms of bodily self-consciousness. *Nat. Rev. Neurosci.* 13 556–571. 10.1038/nrn3292 22805909

[B7] Botvinick M. Cohen J. (1998). Rubber hands ‘feel’ touch that eyes see. *Nature* 391 756–756. 10.1038/35784 9486643

[B8] Boucher R. Bryden M. P. Roy E. A. (1996). *A Construct Approach to the Assessment of Handedness.* Available online at: http://www.ucl.ac.uk/medical-education/other-studies/laterality/laterality-questionnaires/BoucherBrydenAndRoy-1996-WaterlooHandednessQuestionnaire-Revised.pdf (accessed December 1, 2018).

[B9] Braun N. Debener S. Spychala N. Bongartz E. Sörös P. Müller H. H. O. (2018). The senses of agency and ownership: a review. *Front. Psychol.* 9:535. 10.3389/fpsyg.2018.00535 29713301 PMC5911504

[B10] Burin D. Kilteni K. Rabuffetti M. Slater M. Pia L. (2019). Body ownership increases the interference between observed and executed movements. *PLoS One* 14:e0209899. 10.1371/journal.pone.0209899 30605454 PMC6317814

[B11] Cuppone A. V. Squeri V. Semprini M. Masia L. Konczak J. (2016). Robot-assisted proprioceptive training with added vibro-tactile feedback enhances somatosensory and motor performance. *PLoS One* 11:e0164511. 10.1371/journal.pone.0164511 27727321 PMC5058482

[B12] de Mello Monteiro C. B. Massetti T. da Silva T. D. van der Kamp J. de Abreu L. C. Leone C. (2014). Transfer of motor learning from virtual to natural environments in individuals with cerebral palsy. *Res. Dev. Disabil.* 35 2430–2437. 10.1016/j.ridd.2014.06.006 24981192

[B13] Ehrsson H. H. Holmes N. P. Passingham R. E. (2005). Touching a rubber hand: feeling of body ownership is associated with activity in multisensory brain areas. *J. Neurosci. Off. J. Soc. Neurosci.* 25 10564–10573. 10.1523/JNEUROSCI.0800-05.2005 16280594 PMC1395356

[B14] Ehrsson H. H. Spence C. Passingham R. E. (2004). That’s my hand! Activity in premotor cortex reflects feeling of ownership of a limb. *Science* 305 875–877. 10.1126/science.1097011 15232072

[B15] Feng J. Kim J. Luu W. Palmisano S. (2019). “Method for estimating display lag in the Oculus Rift S and CV1,” in *In Proceedings of the SIGGRAPH Asia 2019 Posters (SA ‘19). Association for Computing Machinery*, Vol. 39 New York, NY, 1–2. 10.1145/3355056.3364590

[B16] Ferri F. Chiarelli A. M. Merla A. Gallese V. Costantini M. (2013). The body beyond the body: expectation of a sensory event is enough to induce ownership over a fake hand. *Proc. Biol. Sci.* 280:20131140. 10.1098/rspb.2013.1140 23804622 PMC3712451

[B17] Gassert R. Dietz V. (2018). Rehabilitation robots for the treatment of sensorimotor deficits: a neurophysiological perspective. *J. Neuroeng. Rehabil.* 15:46. 10.1186/s12984-018-0383-x 29866106 PMC5987585

[B18] Gentile G. Guterstam A. Brozzoli C. Ehrsson H. H. (2013). Disintegration of multisensory signals from the real hand reduces default limb self-attribution: an fMRI study. *J. Neurosci.* 33 13350–13366. 10.1523/JNEUROSCI.1363-13.2013 23946393 PMC3742923

[B19] Grechuta K. Guga J. Maffei G. Ballester B. R. Verschure P. F. M. J. (2017). Visuotactile integration modulates motor performance in a perceptual decision-making task. *Sci. Rep.* 7:3333. 10.1038/s41598-017-03488-0 28611387 PMC5469742

[B20] Grechuta K. Ulysse L. Rubio Ballester B. Verschure P. F. M. J. (2019). Self beyond the body: action-driven and task-relevant purely distal cues modulate performance and body ownership. *Front. Hum. Neurosci.* 13:91. 10.3389/fnhum.2019.00091 30949038 PMC6435571

[B21] Hecht D. Reiner M. (2009). Sensory dominance in combinations of audio, visual and haptic stimuli. *Exp. Brain Res.* 193 307–314. 10.1007/s00221-008-1626-z 18985327

[B22] Hoaglin D. C. (2003). John W. Tukey and data analysis. *Stat. Sci* 18 311–318. 10.1214/ss/1076102418

[B23] Isayama R. Vesia M. Jegatheeswaran G. Elahi B. Gunraj C. A. Cardinali L. (2019). Rubber hand illusion modulates the influences of somatosensory and parietal inputs to the motor cortex. *J. Neurophysiol.* 121 563–573. 10.1152/jn.00345.2018 30625001

[B24] Jensen A. R. Rohwer W. D. (1966). The stroop color-word test: a review. *Acta Psychol. (Amst.)* 25 36–93. 10.1016/0001-6918(66)90004-7 5328883

[B25] Kalckert A. Ehrsson H. H. (2012). Moving a rubber hand that feels like your own: a dissociation of ownership and agency. *Front. Human Neurosci.* 6:40. 10.3389/fnhum.2012.00040 22435056 PMC3303087

[B26] Kalckert A. Ehrsson H. H. (2014). The moving rubber hand illusion revisited: comparing movements and visuotactile stimulation to induce illusory ownership. *Conscious Cogn.* 26 117–132. 10.1016/j.concog.2014.02.003 24705182

[B27] Kerns J. G. Cohen J. D. MacDonald A. W. Cho R. Y. Stenger V. A. Carter C. S. (2004). Anterior cingulate conflict monitoring and adjustments in control. *Science* 303 1023–1026. 10.1126/science.1089910 14963333

[B28] Kilteni K. Groten R. Slater M. (2012). The sense of embodiment in virtual reality. *Presence Teleoper. Virtual Env.* 21 373–387. 10.1162/PRES_a_00124

[B29] Kim J. Luu W. Palmisano S. (2020). Multisensory integration and the experience of scene instability, presence and cybersickness in virtual environments. *Comput. Hum. Behav.* 113:106484. 10.1016/j.chb.2020.106484

[B30] Kokkinara E. Slater M. (2014). Measuring the effects through time of the influence of visuomotor and visuotactile synchronous stimulation on a virtual body ownership illusion. *Perception* 43 43–58. 10.1068/p7545 24689131

[B31] Lee I. S. Chae Y. (2016). Neural network underlying recovery from disowned bodily states induced by the rubber hand illusion. *Neural Plast.* 2016:8307175. 10.1155/2016/8307175 28116171 PMC5223049

[B32] Lenggenhager B. Tadi T. Metzinger T. Blanke O. (2007). Video ergo sum: manipulating bodily self-consciousness. *Science* 317 1096–1099. 10.1126/science.1143439 17717189

[B33] Longo M. R. Schüür F. Kammers M. P. M. Tsakiris M. Haggard P. (2008). What is embodiment? A psychometric approach. *Cognition* 107 978–998. 10.1016/j.cognition.2007.12.004 18262508

[B34] Ma K. Hommel B. (2013). The virtual-hand illusion: effects of impact and threat on perceived ownership and affective resonance. *Front. Psychol.* 4:604. 10.3389/fpsyg.2013.00604 24046762 PMC3764400

[B35] MacLeod C. M. (1991). Half a century of research on the Stroop effect: an integrative review. *Psychol. Bull.* 109 163–203. 10.1037/0033-2909.109.2.163 2034749

[B36] Maravita A. Spence C. Driver J. (2003). Multisensory integration and the body schema: close to hand and within reach. *Curr. Biol.* 13 R531–R539. 10.1016/s0960-9822(03)00449-4 12842033

[B37] Marchal-Crespo L. Reinkensmeyer D. J. (2008). Haptic guidance can enhance motor learning of a steering task. *J. Mot. Behav.* 40 545–556. 10.3200/JMBR.40.6.545-557 18980907

[B38] Marchal-Crespo L. Reinkensmeyer D. J. (2009). Review of control strategies for robotic movement training after neurologic injury. *J. Neuroeng. Rehabil.* 6:20. 10.1186/1743-0003-6-20 19531254 PMC2710333

[B39] Maselli A. Slater M. (2013). The building blocks of the full body ownership illusion. *Front. Hum. Neurosci.* 7:83. 10.3389/fnhum.2013.00083 23519597 PMC3604638

[B40] Naber M. Vedder A. Brown S. B. R. E. Nieuwenhuis S. (2016). Speed and lateral inhibition of stimulus processing contribute to individual differences in stroop-task performance. *Front. Psychol.* 7:822. 10.3389/fpsyg.2016.00822 27313555 PMC4887505

[B41] Özen Ö. Buetler K. A. Marchal-Crespo L. (2021). Promoting motor variability during robotic assistance enhances motor learning of dynamic tasks. *Front. Neurosci*. 14:600059. 10.3389/fnins.2020.600059 33603642 PMC7884323

[B42] Palmisano S. Allison R. S. Kim J. (2020). Cybersickness in head-mounted displays is caused by differences in the user’s virtual and physical head pose. *Front. Virtual Real.* 1:587698. 10.3389/frvir.2020.587698 PMC9761034 36567954

[B43] Palmisano S. Mursic R. Kim J. (2017). Vection and cybersickness generated by head-and-display motion in the Oculus Rift. *Displays* 46 1–8. 10.1016/j.displa.2016.11.001

[B44] Pataky T. C. (2012). One-dimensional statistical parametric mapping in Python. *Comput. Methods Biomech. Biomed. Eng.* 15 295–301. 10.1080/10255842.2010.527837 21756121

[B45] Perez-Marcos D. Bieler-Aeschlimann M. Serino A. (2018). Virtual reality as a vehicle to empower motor-cognitive neurorehabilitation. *Front. Psychol.* 9:2120. 10.3389/fpsyg.2018.02120 30450069 PMC6224455

[B46] Perez-Marcos D. Sanchez-Vives M. V. Slater M. (2012). Is my hand connected to my body? The impact of body continuity and arm alignment on the virtual hand illusion. *Cogn. Neurodyn.* 6 295–305. 10.1007/s11571-011-9178-5 24995046 PMC4079845

[B47] Petkova V. I. Ehrsson H. H. (2008). If I were you: perceptual illusion of body swapping. *PLoS One* 3:e3832. 10.1371/journal.pone.0003832 19050755 PMC2585011

[B48] Petkova V. I. Khoshnevis M. Ehrsson H. H. (2011). The perspective matters! Multisensory integration in ego-centric reference frames determines full-body ownership. *Front. Psychol.* 2:35. 10.3389/fpsyg.2011.00035 21687436 PMC3108400

[B49] Pritchard S. C. Zopf R. Polito V. Kaplan D. M. Williams M. A. (2016). Non-hierarchical influence of visual form, touch, and position cues on embodiment, agency, and presence in virtual reality. *Front. Psychol.* 7:1649. 10.3389/fpsyg.2016.01649 27826275 PMC5078469

[B50] Rose F. D. Brooks B. M. Rizzo A. A. (2005). Virtual reality in brain damage rehabilitation: review. *Cyberpsychol. Behav.* 8 241–262; discussion 263–271. 10.1089/cpb.2005.8.241 15971974

[B51] Rubo M. Gamer M. (2019). Visuo-tactile congruency influences the body schema during full body ownership illusion. *Conscious. Cogn.* 73:102758. 10.1016/j.concog.2019.05.006 31176847 PMC6694184

[B52] Sanchez-Vives M. V. Spanlang B. Frisoli A. Bergamasco M. Slater M. (2010). Virtual hand illusion induced by visuomotor correlations. *PLoS One* 5:e10381. 10.1371/journal.pone.0010381 20454463 PMC2861624

[B53] Shibuya S. Unenaka S. Ohki Y. (2018a). The relationship between the virtual hand illusion and motor performance. *Front. Psychol.* 9:2242. 10.3389/fpsyg.2018.02242 30515118 PMC6255939

[B54] Shibuya S. Unenaka S. Zama T. Shimada S. Ohki Y. (2018b). Spontaneous imitative movements induced by an illusory embodied fake hand. *Neuropsychologia.* 111 77–84. 10.1016/j.neuropsychologia.2018.01.023 29407592

[B55] Skarbez R. Brooks F. P. Jr. Whitton M. C. (2017). A survey of presence and related concepts. *ACM Comput. Surveys (CSUR)* 50 1–39. 10.1145/3134301

[B56] Slater M. Perez-Marcos D. Ehrsson H. H. Sanchez-Vives M. V. (2008). Towards a digital body: the virtual arm illusion. *Front. Hum. Neurosci.* 2:6. 10.3389/neuro.09.006.2008 18958207 PMC2572198

[B57] Slater M. Spanlang B. Sanchez-Vives M. V. Blanke O. (2010). First person experience of body transfer in virtual reality. *PLoS One* 5:e10564. 10.1371/journal.pone.0010564 20485681 PMC2868878

[B58] Tsakiris M. (2010). My body in the brain: a neurocognitive model of body-ownership. *Neuropsychologia* 48 703–712. 10.1016/j.neuropsychologia.2009.09.034 19819247

[B59] Tsakiris M. Haggard P. (2005). The rubber hand illusion revisited: visuotactile integration and self-attribution. *J. Exp. Psychol. Hum. Percept. Perform.* 31 80–91. 10.1037/0096-1523.31.1.80 15709864

[B60] van Dijk M. T. van Wingen G. A. van Lammeren A. Blom R. M. de Kwaasteniet B. P. Scholte H. S. (2013). Neural basis of limb ownership in individuals with body integrity identity disorder. *PLoS One* 8:e72212. 10.1371/journal.pone.0072212 23991064 PMC3749113

[B61] Weech S. Kenny S. Barnett-Cowan M. (2019). Presence and cybersickness in virtual reality are negatively related: a review. *Front. Psychol.* 10:158. 10.3389/fpsyg.2019.00158 30778320 PMC6369189

[B62] Wenk N. Penalver-Andres J. Palma R. Buetler K. A. Muri R. Nef T. (2019). Reaching in several realities: motor and cognitive benefits of different visualization technologies. *IEEE Int. Conf. Rehabil. Robot. Proc.* 2019 1037–1042. 10.1109/ICORR.2019.8779366 31374766

[B63] Wise S. P. (1985). The primate premotor cortex: past, present, and preparatory. *Annu. Rev. Neurosci.* 8 1–19. 10.1146/annurev.ne.08.030185.000245 3920943

[B64] Witmer B. G. Singer M. J. (1998). Measuring presence in virtual environments: a presence questionnaire. *Presence Teleoperat. Virtual Environ.* 7 225–240. 10.1162/105474698565686

[B65] Zeller D. Friston K. J. Classen J. (2016). Dynamic causal modeling of touch-evoked potentials in the rubber hand illusion. *Neuroimage* 138 266–273. 10.1016/j.neuroimage.2016.05.065 27241481

[B66] Zeller D. Gross C. Bartsch A. Johansen-Berg H. Classen J. (2011). Ventral premotor cortex may be required for dynamic changes in the feeling of limb ownership: a lesion study. *J. Neurosci.* 31 4852–4857. 10.1523/JNEUROSCI.5154-10.2011 21451023 PMC3119817

