# Supplementary material for: Congruency of Information Rather Than Body Ownership Enhances Motor Performance in Highly Embodied Virtual Reality
Source: Front Neurosci. 2021 Jul 2;15:678909. doi: 10.3389/fnins.2021.678909 (PMC8291288; doi:10.3389/fnins.2021.678909)
Supplement: Supplementary file 1 [file Table_1.pdf]

Supplementary Material S1

Task instructions

1 English version

1.1 Task 1 (sensorimotor decision-making task):

Instructions were adapted from Grechuta et al. (2017) with two changes: 1) Participants were told to be as fast as possible, and 2) to not prime participants, we avoided “virtual” and “real hand”. Instead, we pointed up “feel” or “see” to make clear, whether they had to react to the visual or haptic information. For the congruent condition, neither the visual nor the haptic modality was mentioned.

CONGRUENT:
Please look at the right hand during the whole task. You will see and/or feel a brush stroking the fingers. Press the red button with your left index finger, as soon as the **index finger** of the right hand is stroked. Press as fast as possible.
Please ask if something is unclear to you.
Press the red button to start.

VISUAL INCONGRUENT:
Please look at the right hand during the whole task. You will see and/or feel a brush stroking the fingers. Press the red button with your left index finger, as soon as you **see** that the brush strokes the **index finger** of the right hand. Press as fast as possible.
Please ask if something is unclear to you.
Press the red button to start.

HAPTIC INCONGRUENT:
Please look at the right hand during the whole task. You will see and/or feel a brush stroking the fingers. Press the red button with your left index finger, as soon as you **feel** that the brush strokes the **index finger** of the right hand. Press as fast as possible.
Please ask if something is unclear to you.
Press the red button to start.

VISUAL ONLY:
Please look at the right hand during the whole task. You will see and/or feel a brush stroking the fingers. Press the red button with your left index finger, as soon as you **see** that the brush strokes the **index finger** of the right hand. Press as fast as possible.
Please ask if something is unclear to you.
Press the red button to start.

HAPTIC ONLY:
Please look at the right hand during the whole task. You will see and/or feel a brush stroking the fingers. Press the red button with your left index finger, as soon as you **feel** that the brush strokes the index finger of the right hand. Press as fast as possible.

Please ask if something is unclear to you.
Press the red button to start.

1.2 Transition: (shown to all conditions)

You will be in a dark room for a short time. Lift your right hand when you are instructed to do so. A box will be placed on the table. Please take then the controller. Try to hold it as you can see in the picture below. Please keep this position during the next task. When you come back to the living room, place the right hand on the box.

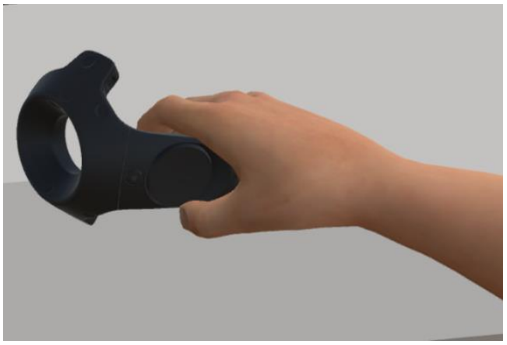

Supplementary Figure 1. Picture showing how to hold the controller with the right hand.

1.3 Task 2a (motor task):

CONGRUENT:
You will again see and/or feel a brush stroking the fingers. Sometimes a butterfly will appear and fly away. It will leave a path with stars behind. Follow this path with the same finger, on which the brush was when the butterfly appeared. Follow the path as fast and precise as possible. The stars on the path will disappear when you pass them with the correct finger. Move continuously: if you miss a star, do not go back.
The task is completed as soon as you touch the butterfly with the correct finger and it disappears. Put the hand then back in the green sphere.

VISUAL INCONGRUENT:
You will again see and/or feel a brush stroking the fingers. Sometimes a butterfly will appear and fly away. It will leave a path with stars behind. Follow this path with the same finger, on which you **saw** the brush when the butterfly appeared. Follow the path as fast and precise as possible. The stars on the path will disappear when you pass them with the correct finger. Move continuously: if you miss a star, do not go back.
The task is completed as soon as you touch the butterfly with the correct finger and it disappears. Put the hand then back in the green sphere

HAPTIC INCONGRUENT:
You will again see and/or feel a brush stroking the fingers. Sometimes a butterfly will appear and fly away. It will leave a path with stars behind. Follow this path with the same finger, on which you **felt** the brush when the butterfly appeared. Follow the path as fast and precise as possible. The stars on the path will disappear when you pass them with the correct finger. Move continuously: if you miss a star, do not go back.

The task is completed as soon as you touch the butterfly with the correct finger and it disappears. Put the hand then back in the green sphere.

VISUAL ONLY:
You will again see and/or feel a brush stroking the fingers. Sometimes a butterfly will appear and fly away. It will leave a path with stars behind. Follow this path with the same finger, on which you **saw** the brush when the butterfly appeared. Follow the path as fast and precise as possible. The stars on the path will disappear when you pass them with the correct finger. Move continuously: if you miss a star, do not go back.
The task is completed as soon as you touch the butterfly with the correct finger and it disappears. Put the hand then back in the green sphere.

HAPTIC ONLY:
You will again see and/or feel a brush stroking the fingers. Sometimes a butterfly will appear and fly away. It will leave a path with stars behind. Follow this path with the same finger, on which you **felt** the brush when the butterfly appeared. Follow the path as fast and precise as possible. The stars on the path will disappear when you pass them with the correct finger. Move continuously: if you miss a star, do not go back.
The task is completed as soon as you touch the butterfly with the correct finger and it disappears. Put the hand then back in the green sphere.

1.4 Task 2b: (shown to all conditions)

Follow the path with the green marked part of the finger. Example: middle finger.

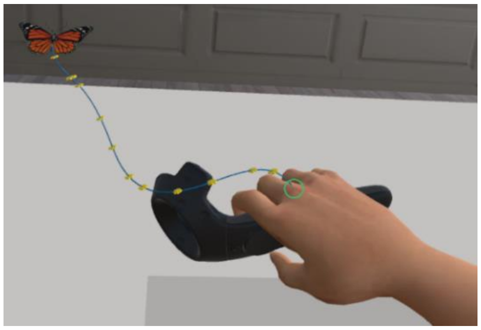

Supplementary Figure 2. Picture of a trial of Task 2 (butterfly task) indicating with which part of the finger the path should be followed.

2 German version

2.1 Task 1 (sensorimotor decision-making task):

CONGRUENT:
Bitte schaue während der ganzen Aufgabe auf die rechte Hand. Du wirst sehen und/oder spüren, wie ein Pinsel die Finger streichelt. Drücke die rote Taste mit deinem linken Zeigefinger, sobald der **Zeigefinger** der rechten Hand gestreichelt wird. Drücke so schnell wie möglich.
Bitte frage nach, falls etwas unklar ist. Drücke die rote Taste, um zu beginnen.

VISUAL INCONGRUENT:
Bitte schaue während der ganzen Aufgabe auf die rechte Hand. Du wirst sehen und/oder spüren, wie ein Pinsel die Finger streichelt. Drücke die rote Taste mit deinem linken Zeigefinger, sobald du **siehst**, dass der Pinsel den **Zeigefinger** der rechten Hand streichelt. Drücke so schnell wie möglich. Bitte frage nach, falls etwas unklar ist. Drücke die rote Taste, um zu beginnen.

HAPTIC INCONGRUENT:
Bitte schaue während der ganzen Aufgabe auf die rechte Hand. Du wirst sehen und/oder spüren, wie ein Pinsel die Finger streichelt. Drücke so schnell wie möglich die rote Taste mit deinem linken Zeigefinger, sobald du **spürst**, dass der Pinsel den **Zeigefinger** der richtigen rechten Hand streichelt. Bitte frage nach, falls etwas unklar ist. Drücke die rote Taste, um zu beginnen.

VISUAL ONLY:
Bitte schaue während der ganzen Aufgabe auf die rechte Hand. Du wirst sehen und/oder spüren, wie ein Pinsel die Finger streichelt. Drücke so schnell wie möglich die rote Taste mit deinem linken Zeigefinger, sobald du **siehst**, dass der Pinsel den **Zeigefinger** der rechten virtuellen Hand streichelt. Bitte frage nach, falls etwas unklar ist. Drücke die rote Taste, um zu beginnen.

HAPTIC ONLY:
Bitte schaue während der ganzen Aufgabe auf die rechte Hand. Du wirst sehen und/oder spüren, wie ein Pinsel die Finger streichelt. Drücke so schnell wie möglich die rote Taste mit deinem linken Zeigefinger, sobald du **spürst**, dass der Pinsel den **Zeigefinger** der richtigen rechten Hand streichelt. Bitte frage nach, falls etwas unklar ist. Drücke die rote Taste, um zu beginnen.

2.2 Transition: (shown to all conditions)

Du wirst für kurze Zeit in einem dunklen Raum sein. Hebe deine rechte Hand, wenn du dazu aufgefordert wirst. Auf den Tisch wird eine Schachtel gelegt. Bitte nimm dann den Controller und versuche ihn so zu halten, wie du auf dem Bild unten siehst. Lege die Hand zurück auf die Schachtel.

2.3 Task 2a (motor task):

CONGRUENT:
Du wirst wieder sehen und/oder spüren, wie ein Pinsel die Finger streichelt. Manchmal wird ein Schmetterling erscheinen und wegfliegen. Er wird eine Spur mit Sternen hinterlassen. Folge dieser Spur mit dem gleichen Finger, auf dem der Pinsel war, als der Schmetterling erschien. Folge der Spur so schnell und genau wie möglich. Die Sterne auf der Spur verschwinden, wenn du sie mit dem richtigen Finger berührst. Mache eine kontinuierliche Bewegung: Wenn du einen Stern verpasst, gehe nicht zurück.
Die Aufgabe ist erfüllt, sobald du den Schmetterling mit dem richtigen Finger berührst und er verschwindet. Lege die Hand dann zurück in die grüne Kugel.

VISUAL INCONGRUENT:
Du wirst wieder sehen und/oder spüren, wie ein Pinsel die Finger streichelt. Manchmal wird ein Schmetterling erscheinen und wegfliegen. Er wird eine Spur mit Sternen hinterlassen. Folge dieser Spur mit dem gleichen Finger, auf dem du den Pinsel **gesehen** hast, als der Schmetterling erschien. Folge der Spur so schnell und genau wie möglich. Die Sterne auf der Spur verschwinden, wenn du sie mit dem richtigen Finger berührst. Mache eine kontinuierliche Bewegung: Wenn du einen Stern

verpasst, gehe nicht zurück.
Die Aufgabe ist erfüllt, sobald du den Schmetterling mit dem richtigen Finger berührst und er verschwindet. Lege die Hand dann zurück in die grüne Kugel.

HAPTIC INCONGRUENT:
Du wirst wieder sehen und/oder spüren, wie ein Pinsel die Finger streichelt. Manchmal wird ein Schmetterling erscheinen und wegfliegen. Er wird eine Spur mit Sternen hinterlassen. Folge dieser Spur mit dem gleichen Finger, auf dem du den Pinsel **gespürt** hast, als der Schmetterling erschien. Folge der Spur so schnell und genau wie möglich. Die Sterne auf der Spur verschwinden, wenn du sie mit dem richtigen Finger berührst. Mache eine kontinuierliche Bewegung: Wenn du einen Stern verpasst, gehe nicht zurück.
Die Aufgabe ist erfüllt, sobald du den Schmetterling mit dem richtigen Finger berührst und er verschwindet. Lege die Hand dann zurück in die grüne Kugel.

VISUAL ONLY:
Du wirst wieder sehen und/oder spüren, wie ein Pinsel die Finger streichelt. Manchmal wird ein Schmetterling erscheinen und wegfliegen. Er wird eine Spur mit Sternen hinterlassen. Folge dieser Spur mit dem gleichen Finger, auf dem du den Pinsel **gesehen** hast, als der Schmetterling erschien. Folge der Spur so schnell und genau wie möglich. Die Sterne auf der Spur verschwinden, wenn du sie mit dem richtigen Finger berührst. Mache eine kontinuierliche Bewegung: Wenn du einen Stern verpasst, gehe nicht zurück.
Die Aufgabe ist erfüllt, sobald du den Schmetterling mit dem richtigen Finger berührst und er verschwindet. Lege die Hand dann zurück in die grüne Kugel.

HAPTIC ONLY:
Du wirst wieder sehen und/oder spüren, wie ein Pinsel die Finger streichelt. Manchmal wird ein Schmetterling erscheinen und wegfliegen. Er wird eine Spur mit Sternen hinterlassen. Folge dieser Spur mit dem gleichen Finger, auf dem du den Pinsel **gespürt** hast, als der Schmetterling erschien. Folge der Spur so schnell und genau wie möglich. Die Sterne auf der Spur verschwinden, wenn du sie mit dem richtigen Finger berührst. Mache eine kontinuierliche Bewegung: Wenn du einen Stern verpasst, gehe nicht zurück.
Die Aufgabe ist erfüllt, sobald du den Schmetterling mit dem richtigen Finger berührst und er verschwindet. Lege die Hand dann zurück in die grüne Kugel.

2.4 Task 2b: (shown to all conditions)

Folge der Spur mit dem grün markierten Teil des Fingers. Beispiel: Mittelfinger.
