# Supplementary material for: Congruency of Information Rather Than Body Ownership Enhances Motor Performance in Highly Embodied Virtual Reality
Source: Front Neurosci. 2021 Jul 2;15:678909. doi: 10.3389/fnins.2021.678909 (PMC8291288; doi:10.3389/fnins.2021.678909)
Supplement: Supplementary file 2 [file Table_2.pdf]

frontiers

Supplementary Material S2

Reports of statistical analyses

BLOCK 1

**One-way ANOVA**	Control items<br>Baseline questionnaire	$F(4,45) = 0.36$	$p = 0.84$
**One-way ANOVA**	Control items<br>Embodiment questionnaire	$F(4,45) = 0.51$	$p = 0.73$
**One-way ANOVA**	BO-Task<br>Embodiment questionnaire	$F(4,45) = 1.27$	$p = 0.30$
**Correlation**	GSR-mean and BO-Task	$r(46) = -0.22$	$p = 0.13$
**Correlation**	GSR-peak and BO-Task	$r(46) = -0.25$	$p = 0.08$
**One-way ANOVA**	Reaction time Baseline	$F(4,45) = 0.71$	$p = 0.59$
**One-way ANOVA**	False positives in Task 1	$F(4,45) = 1.03$	$p = 0.40$
**One-way ANOVA**	False negatives in Task 1	$F(4,45) = 2.03$	$p = 0.11$
**Two-way ANOVA**	Reaction times Task 1<br>Main effect Trial	$F(4,45) = 1.16$	$p = 0.32$
**Correlation**	Reaction times and BO-Task	$r(48) = -0.16$	$p = 0.26$

BLOCK 2

**One-way ANOVA**	Body ownership<br>Baseline questionnaire	$F(4,45) = 0.57$	$p = 0.69$
**One-way ANOVA**	Control items<br>Baseline questionnaire	$F(4,45) = 1.51$	$p = 0.22$
**One-way ANOVA**	BO-Task<br>Embodiment questionnaire	$F(4,45) = 0.73$	$p = 0.58$
**One-way ANOVA**	BO-Diff	$F(4,45) = 1.59$	$p = 0.19$
**One-way ANOVA**	Agency<br>Embodiment questionnaire	$F(4,45) = 0.84$	$p = 0.51$
**Correlation**	GSR-mean and BO-Task	$r(47) = -0.10$	$p = 0.48$
**Correlation**	GSR-peak and BO-Task	$r(47) = -0.07$	$p = 0.72$

**Two-way ANOVA**	Completion time Task 2<br>Main effect Trial<br>Interaction (Condition x Trial)	 *F*(2,90) = 0.54<br>*F*(8,90) = 0.50	 *p* = 0.54<br>*p* = 0.80
**Two-way ANOVA**	Accuracy Task 2<br>Main effect Condition<br>Interaction (Condition x Trial)	 *F*(4,45) = 0.25<br>*F*(8,90) = 1.26	 *p* = 0.91<br>*p* = 0.28

ACROSS TASKS

**Two-way ANOA**	BO-Baseline<br>Main effect Condition<br>Interaction (Condition x Block)	 *F*(4,45) = 1.76<br>F(8,90) = 2.25	 *p* = 0.15<br>*p* = 0.08
**Two-way ANOVA**	BO-Task<br>Main effect Condition<br>Interaction (Condition x Block)	 *F*(4,45) = 0.91<br>*F*(8,90) = 1.91	 *p* = 0.47<br>*p* = 0.13
**Correlation**	Reaction times Task 1 and accuracy Task 2	*r*(48) = 0.09	*p* = 0.52
